# Designing and evaluating LLM-driven coaching agents for VR public speaking practice

**DOI:** 10.3389/fpsyg.2026.1886780

**Published:** 2026-07-08

**Authors:** Enyao Chang, Xiaoping Che, Jianing Zhang

**Affiliations:** 1 School of Software Engineering, Beijing Jiaotong University, Beijing, China; 2 School of Cyberspace Science and Technology, Beijing Jiaotong University, Beijing, China

**Keywords:** affective computing, embodied conversational agents, human–computer interaction, large language models, public speaking anxiety, public speaking practice, virtual reality, VR training

## Abstract

**Introduction:**

Public speaking practice in Virtual Reality (VR) provides an immersive and controllable setting, yet many existing systems offer limited adaptive guidance beyond exposure itself. Recent advances in Large Language Models (LLMs) create opportunities for designing coaching interactions that are personalized, context-aware, and socially supportive.

**Methods:**

We designed a VR public speaking system in which embodied virtual judges delivered spoken and textual, performance-contingent coaching based on automatic speech recognition, speech synthesis, gaze-related cues, and structured prompting with ERNIE Bot. We evaluated the system in a two-arm randomized controlled user study with 120 participants, with 60 participants assigned to the adaptive coaching condition and 60 to an attention-matched generic feedback control condition. Participants completed two rounds of VR speeches with repeated pre- and post-speech self-report assessments, followed by matched follow-up sessions at one week and one month.

**Results:**

Compared with attention-matched generic feedback, the adaptive coaching condition was associated with larger reductions in self-reported state anxiety and PRCA-derived public speaking anxiety appraisals across repeated speaking rounds. At follow-up sessions conducted in the same VR environment without additional coaching, lower self-reported anxiety remained observable in the experimental group. Qualitative feedback suggested that participants experienced the agents as supportive, useful, and lower-pressure, while also noting limitations in realism, speech prosody, and interaction richness.

**Discussion:**

The findings provide preliminary evidence that embodied LLM-based coaching can support anxiety-aware VR public speaking practice. However, because the study relied primarily on self-report, did not include physiological outcomes or blinded external performance ratings, and involved repeated exposure to the same VR context, the follow-up findings should be interpreted as maintenance within the VR practice setting rather than evidence of stable trait change, objective performance improvement, or real-world transfer. Future studies should examine whether these self-reported gains generalize to real-world speaking contexts and externally rated public speaking performance.

## 1 Introduction

Public speaking is common in educational, professional, and social life, yet many people experience anxiety when speaking before an audience. Public Speaking Anxiety (PSA) is one of the most common forms of social anxiety, affecting an estimated 15%–30% of the population (Tejwani et al., 2016). PSA can undermine verbal expression, self-confidence, and participation in communication-intensive tasks, making it difficult for speakers to practice effectively and improve over time. In higher education in particular, students are frequently required to deliver classroom presentations, but systematic and personalized speaking support remains limited (Kuit, 2024). Prior work has also shown that PSA can be reflected in both self-report measures and physiological responses, suggesting that public speaking support should address not only speech performance but also users' lived experience of stress during the task (Radomsky and van den Hout, 2004; Carrillo et al., 2001).

Virtual Reality (VR) has emerged as a promising medium for public speaking practice because it can simulate socially evaluative situations while maintaining experimental control over audience behavior, environmental atmosphere, and task intensity (Chollet et al., 2015; Abbas et al., 2023; Slater et al., 1999). A central concept in VR is *presence*, commonly understood as the subjective sense of being located in a virtual environment and experiencing virtual events as plausible or meaningful (Slater, 2009). In VR public speaking, presence is especially important because speakers may respond emotionally to virtual audiences even when they know that the audience is computer-generated (Slater et al., 1999). Prior studies have shown that VR-based public speaking systems can reduce anxiety, improve speaking-related confidence, and, in some training contexts, support persuasive and charismatic delivery (Lim et al., 2023; Vafadar, 2013; Frisby et al., 2020; Valls-Rates et al., 2023). Recent validation work comparing real and virtual audiences also suggests that VR public speaking tasks can elicit meaningful anxiety-, voice-, and fluency-related responses (Bettahi et al., 2026). Together, this work indicates that VR public speaking practice is not only a simulation problem, but also an interaction design problem: users' responses are shaped by how the audience behaves, how evaluative pressure is represented, and how feedback is delivered (Girondini et al., 2023, 2024).

Many VR public speaking systems still rely on predefined or passive practice modes. They place users in front of an audience, but offer limited adaptive support for what the speaker says, how the speech is delivered, or how the speaker feels during the task. Yet public speaking practice often requires more than repeated exposure. Speakers also need feedback that is timely, actionable, and responsive to their current state. Prior work on Conversational Agents (CAs) suggests that supportive voice-based interaction can help users reframe negative self-talk and feel more comfortable before speaking (Wang et al., 2020). This highlights the role of social presence, supportive language, and perceived interpersonal closeness in anxiety-related interactive systems (Powers and Kiesler, 2006; Kumar and Benbasat, 2002).

Large Language Models (LLMs) further expand this design space by enabling feedback that can be adapted to users' speech content, delivery patterns, and interaction context. Recent LLM-based public speaking systems have explored configurable audiences, LLM-generated personas, and chatbot-based question-and-answer rehearsal (Park and Choi, 2023; Min and Jeong, 2024; La et al., 2025). These systems demonstrate the promise of adaptive AI support for public speaking practice. However, many existing systems focus primarily on audience simulation, post-practice comments, or Q&A rehearsal rather than anxiety-aware coaching delivered by embodied agents within the speaking scene itself. This leaves an open design question: how can LLM-driven feedback be embedded in immersive VR public speaking practice in a way that remains adaptive and personalized, while also preserving psychological safety during a socially evaluative task?

In this work, we present the design and evaluation of *LLM-driven coaching agents* for VR public speaking practice. Our system embeds three embodied virtual judges in a classroom-like VR environment and combines automatic speech recognition, text-to-speech synthesis, gaze-related cues, and structured language generation to deliver performance-contingent coaching. We intentionally use judge-like avatars rather than purely coach-like avatars because PSA is closely tied to social evaluation; removing the evaluative role altogether could reduce the ecological plausibility of the speaking task. At the same time, the judges are not designed to intensify pressure or deliver harsh assessment. Instead, their verbal and nonverbal behaviors are constrained to remain supportive, brief, non-shaming, and practice-oriented. In this way, the system aims to preserve the realism of speaking before an audience while providing actionable guidance that users can apply in the next speaking round.

To examine the value of this design, we conducted a two-arm randomized controlled user study with repeated speeches and short-term follow-ups. Participants completed two rounds of VR public speaking, with repeated anxiety assessments before and after each round, and both groups returned for one-week and one-month follow-up sessions in the same VR practice context. In addition to self-reported anxiety outcomes, we examined how users experienced the embodied coaching agents as interactive support in VR. Based on this design, we address the following research questions:

- **RQ1:** How does interactive, performance-contingent coaching compare with attention-matched generic feedback in shaping self-reported anxiety trajectories during repeated VR public speaking practice?
- **RQ2:** How do users perceive and experience *LLM-driven coaching agents* embedded in VR public speaking practice?
- **RQ3:** To what extent are self-reported changes observed after initial practice also observable at 1-week and 1-month follow-up sessions conducted in the same VR context without additional coaching?

Our work makes three main contributions. First, we present the design of a VR public speaking system that integrates embodied *LLM-driven coaching agents* with multimodal interaction components for adaptive feedback. Second, we provide a controlled user study evaluating self-reported anxiety outcomes and users' experience of the coaching interaction during repeated VR public speaking practice. Third, we contribute design insights for building supportive LLM-based coaching interactions in socially evaluative VR settings, with implications for future human–computer interaction research on embodied agents, adaptive feedback, and anxiety-aware practice systems.

## 2 Related work

This section reviews prior work on public speaking anxiety, VR-based public speaking practice and intervention, conversational and embodied agents for anxiety-related support, and recent LLM-driven public speaking systems. We use this review to clarify the gap addressed by the present study: embedding embodied LLM-driven coaching agents within an immersive, socially evaluative VR speaking scene.

### 2.1 Public speaking anxiety

Public speaking anxiety (PSA) is a common situational form of social anxiety. It includes affective discomfort, cognitive worry, avoidance tendencies, and physiological responses such as increased heart rate, trembling voice, and difficulty concentrating when speaking before an audience (Harris et al., 2002; Lee et al., 2002). PSA can affect classroom presentations, interviews, workplace communication, and broader participation in communication-intensive tasks. Prior survey work reported that more than 40% of college graduates were afraid to express their ideas in public and believed that this fear harmed their communication and leadership development (Harris et al., 2002).

PSA is often discussed in relation to both trait and state anxiety. Trait anxiety refers to a relatively stable tendency toward tension, worry, or avoidance, whereas state anxiety refers to temporary arousal caused by a specific speaking task (Poeschl, 2017). This distinction is important for interpreting short VR interventions. A brief practice session may reduce state anxiety or change users' self-appraisals of public speaking, but it should not be interpreted as changing stable trait anxiety. Accordingly, in the present study, we distinguish acute state anxiety from repeated PRCA-derived public speaking anxiety appraisals.

PSA can be measured through self-report scales, physiological signals, and behavioral indicators. Prior work has used measures such as heart rate, electrodermal activity, voice, fluency, and gaze-related behavior to examine anxiety responses during public speaking tasks (Carrillo et al., 2001; Bettahi et al., 2026; Wechsler et al., 2021; Rubin et al., 2022). Avoidance is also central to PSA: people with high PSA may withdraw from speaking opportunities or simplify what they want to say, which can reinforce fear over time (MacIntyre et al., 1997). Prior work also notes developmental and cultural-context differences. For example, younger students and speakers in contexts that place stronger emphasis on social evaluation, face concerns, or group harmony may report greater vulnerability to PSA (Lee et al., 2002; Kumar et al., 2017).

### 2.2 Virtual reality in PSA intervention

VR is useful for PSA research and intervention because it can recreate public speaking situations under controllable, repeatable, and safe conditions (Finn et al., 2009; Premkumar et al., 2021; Slater et al., 1999). Compared with conventional rehearsal or *in vivo* exposure, VR allows researchers and designers to manipulate audience size, gaze, facial expression, feedback, and environmental intensity while keeping the speaking task consistent across participants. A meta-analysis found that VR training can reduce public speaking anxiety and support speaking-related outcomes (Lim et al., 2023). Other work has shown that VR-assisted public speaking training may also improve persuasiveness, charisma, and speaking confidence in some educational contexts (Valls-Rates et al., 2023; Frisby et al., 2020).

The effects of VR-based public speaking practice depend strongly on scenario and audience design. Scenes with audience expressions and judgmental gaze can make practice more realistic and may support transfer to later speaking situations (Vafadar, 2013; Wallach et al., 2009). Richer audience feedback can support graded social exposure and real-life performance improvement (Kroczek and Muhlberger, 2023). Audience dynamics have also been shown to shape both physiological arousal and subjective anxiety (Girondini et al., 2023). More recent work further suggests that verbal and nonverbal audience behaviors may affect stress responses in different ways (Girondini et al., 2024). Validation studies comparing real and virtual audiences indicate that VR public speaking tasks can elicit meaningful anxiety-, voice-, and fluency-related responses (Bettahi et al., 2026).

Together, these studies show that the virtual audience should not be treated as a passive background element. Audience behavior, social presence, and feedback delivery are part of the intervention itself. However, much of the prior VR work has focused on exposure, audience simulation, or predefined feedback. Less attention has been given to how adaptive coaching can be delivered by embodied agents during immersive public speaking practice while maintaining psychological safety.

### 2.3 Conversational and embodied agents for anxiety-related support

Conversational agents (CAs) have been used for anxiety regulation and mental-health support, especially through language-based interaction. Voice-based coaching can help users reframe negative self-talk and feel more comfortable before public speaking (Wang et al., 2020). Other systems, such as Woebot and Shim, have been used to support users with anxiety or depressive symptoms through structured conversational interaction, self-disclosure, and engagement (Fitzpatrick et al., 2017; Ly et al., 2017). Recent work also supports chatbot-delivered cognitive-behavioral components for university populations (Yokotani et al., 2025).

Embodied Virtual Agents (EVAs) add visual embodiment, social cues, and a stronger sense of interpersonal presence (Bickmore et al., 2010; Rizzo et al., 2016b). Prior work has explored virtual agents and virtual therapists for anxiety, depression, PTSD, healthcare support, and clinical interviewing (Mitrut et al., 2021; Ring et al., 2016; Rizzo et al., 2016a). In public speaking, a virtual coach has been used to support non-native English speakers, with reported reductions in anxiety and improvements in confidence (Trinh et al., 2015). These findings suggest that agents can provide emotional and practical support, but many existing systems operate outside an immersive public speaking scene or provide support that is not closely tied to the speaker's ongoing performance.

### 2.4 LLM-driven public speaking interaction

LLMs extend conversational and embodied-agent research by enabling feedback that can be adapted to speech content, user intent, and interaction context. In public speaking, *AudiLens* provides configurable LLM-generated audiences for public speech practice (Park and Choi, 2023). LLM-generated personas have also been used for VR question-and-answer training (Min and Jeong, 2024). In another related direction, an LLM-based chatbot training application has been explored for alleviating graduate students' public-speaking anxiety during Q&A sessions (La et al., 2025). Beyond public speaking, LLM-based systems have been used for classroom debate, creator feedback, audience-persona simulation, and stress-management practice (Zhang et al., 2025; Choi et al., 2025; Fang et al., 2025).

These studies show the promise of LLMs for adaptive, persona-based, and context-sensitive interaction. At the same time, they also raise open questions about timing, realism, user trust, and safety. In public speaking practice, feedback must be not only relevant but also appropriately brief, actionable, and non-shaming. Moreover, many existing LLM-based public speaking systems emphasize audience generation, Q&A rehearsal, or post-task comments, whereas fewer studies examine embodied LLM-driven coaching agents that are embedded in the speaking scene itself and evaluated through a controlled VR user study.

### 2.5 Research gap

Prior work shows that VR can support public speaking practice, that virtual audience behavior shapes anxiety and presence, and that conversational or embodied agents can provide supportive interaction. Recent LLM-based systems further demonstrate the potential of configurable audiences, persona-based simulation, and adaptive feedback. However, several gaps remain. First, relatively few systems integrate LLM-driven feedback with embodied agents inside an immersive VR public speaking scene. Second, existing systems often focus on exposure, audience behavior, or Q&A rehearsal rather than performance-contingent coaching that responds to the speaker's own content and delivery cues. Third, limited work has evaluated such systems using an attention-matched control condition while also examining users' perceptions of the agents as social and supportive interfaces.

Our work addresses these gaps by designing and evaluating *LLM-driven coaching agents* embodied as virtual judges in a VR classroom. Rather than using LLMs only for audience simulation or generic post-practice comments, we examine whether embodied LLM-based feedback can provide concise, supportive, and performance-contingent coaching within a socially evaluative VR practice context. This framing also keeps the interpretation cautious: the present study evaluates the combined coaching system rather than isolating a single mechanism such as novelty, personalization, embodiment, or repeated exposure.

## 3 System description

This section introduces the system setup, agent design, VR environment, and questionnaire measures. The system is designed as a supportive VR public speaking practice environment rather than as a clinical treatment tool. Its goal is to provide structured, psychologically safe, and performance-contingent coaching during repeated public speaking practice.

### 3.1 Agent design

We embed *LLM-driven coaching agents*, embodied as virtual judges, in a classroom-like VR public speaking environment. The judge role was selected because public speaking anxiety is closely tied to social evaluation. A purely coach-like avatar could reduce the evaluative character of the task and weaken ecological plausibility. At the same time, the agents are not designed to intensify pressure or deliver harsh judgment. Their verbal and nonverbal behavior is constrained to remain supportive, brief, non-shaming, and practice-oriented.

Each agent receives three types of contextual input: (i) an automatic speech recognition (ASR) transcript, (ii) a time series of eye-contact frequency (ECF; Section 3.1.7), and (iii) optional slide or outline text when available. These inputs are used to generate structured coaching comments during the debrief segment between two speech rounds. The design follows recent work on persona-based coaching, LLM-generated audience simulation, and interactive rehearsal systems (La et al., 2025; Min and Jeong, 2024; Choi et al., 2025). Table 1 summarizes how the five design principles are operationalized through prompt constraints, output schema rules, and UI timing decisions. Table 2 summarizes the hardware, software, sensing, LLM backend, decoding, and fallback settings used to implement the VR coaching system.

**Table 1:** How the five design principles are instantiated as prompt constraints, output schema rules, and UI timing decisions.

Principle	Prompt constraint/schema field	UI/timing	Example snippet
Acknowledge and normalize	Require an initial validation sentence; populate anxiety_cues_noticed with evidence-based observations only.	Debrief starts with a short validation sentence before any critique.	“It is normal to feel tense before a talk; your pauses are a common stress signal.”
Micro-interventions	micro_interventions must contain 1–2 items; forbid long lists; each item must be an actionable cue.	Display as two bullets; TTS reads at most two cues to avoid overload.	“One slow exhale before your next point; add one signpost phrase.”
Strengths-first	strengths must be non-empty and appear first in the JSON; disallow negative wording inside strengths.	TTS always reads strengths first to reinforce self-efficacy.	“Your opening was clear and your pacing improved in the last 20 s.”
State then skill	Enforce ordering: a state-regulation cue appears before content_tweak; keep content_tweak to one main fix.	If elevated tension is inferred, the first spoken tip is a grounding cue.	“Before the next example, take one breath; then add a one-sentence takeaway.”
Rehearsable drills	Require next_60s_plan and one_drill; the drill must be time-bounded, usually 30–60 s.	Debrief ends with a ready-to-try drill; users can immediately repeat.	“In 60 s: restate your thesis, then deliver one example with a left–center–right gaze sweep.”

**Table 2:** Implementation details of the VR coaching system.

Component	Specification
VR headset	Oculus Quest Pro
VR engine	Unity 2021.3.34f1 LTS
Operating workstation	Intel Core i7-12700K CPU, NVIDIA RTX 4060 GPU, 32 GB RAM, Windows 11 Pro
Eye tracking	Quest Pro eye tracking, nominal sampling rate 90 Hz; invalid samples discarded
ASR	Unity DictationRecognizer, language setting: en-US
TTS	Baidu WaveNet-style TTS API v4.0
LLM backend	Baidu ERNIE Bot 4.0
Decoding parameters	temperature = 0.25, top_p = 0.70, penalty_score = 1.00, max_completion_tokens = 512
Fallback behavior	Template-based supportive text message; TTS skipped if generation or synthesis fails

#### 3.1.1 Design principles for anxiety-reducing guidance

We operationalize five design principles to provide support without inducing shame or information overload.

- **Acknowledge and normalize**. The agent first validates common PSA reactions and avoids catastrophizing. This principle is intended to reduce the sense that nervousness is unusual or personally disqualifying.
- **Micro-interventions over macro-critique**. The agent provides one or two small, high-yield actions that can be applied immediately, rather than a long list of weaknesses.
- **Strengths-first framing**. The agent surfaces what worked before offering a targeted fix. This ordering is intended to support self-efficacy and reduce the perceived threat of evaluation.
- **State then skill**. The agent first offers a state-regulation cue, such as breathing or grounding, before addressing structure, clarity, or delivery.
- **Rehearsable drills**. The agent ends with a short, time-bounded exercise that the participant can try immediately in the next speaking round.

#### 3.1.2 Mapping principles to system design choices

To translate these principles into a reproducible system, we implemented them at three levels. First, the prompt contains explicit constraints on tone, ordering, and safety. Second, the LLM output must follow a fixed JSON schema with required fields and length limits. Third, the user interface controls the timing and amount of feedback by presenting strengths first, limiting the number of spoken suggestions, and ending the debrief with one ready-to-try drill.

#### 3.1.3 Three anxiety-focused coaching roles

We defined three coaching roles that correspond to a progression from immediate arousal regulation, to speaking-skill support, to mild stress-inoculation practice. The number of roles was chosen as a design decision to keep feedback diverse while avoiding an overly crowded panel. The three roles were not treated as separate experimental conditions. In the present study, all participants in the Experimental group encountered the same three judge roles in the same virtual classroom, and each debrief included one contribution from each role. Therefore, the study evaluates the combined coaching system rather than isolating the independent effect of each level.

- **Level 1: Self-soothing coach**. *Goal*: reduce immediate tension and help the speaker settle into the task. *Style*: warm and concise, with strengths-first feedback, one small intervention, one grounding cue, and at most one brief clarification question.
- **Level 2: Skills coach**. *Goal*: combine light anxiety regulation with support for content and organization. *Style*: encouraging and practice-oriented, with two focused suggestions, such as transitions, examples, or an audience takeaway, and, when needed, one guiding question.
- **Level 3: Stress-inoculation coach**. *Goal*: support brief practice under mild pressure while maintaining a respectful tone. *Style*: direct but nonjudgmental, focusing on one main weakness, followed by a short restatement or rebuttal task, one coping cue, and one drill.

Because the levels were implemented as coordinated roles within one system, no statistical comparison among the three levels is reported. Future studies could isolate these roles to examine whether arousal regulation, skills coaching, or stress-inoculation prompts contribute differently to anxiety reduction and speech improvement.

#### 3.1.4 Prompting strategy with reproducible schemas

To keep the feedback format stable across agents, each judge uses a role- and level-specific prompt with explicit inputs and a fixed JSON schema. The returned JSON is rendered in two forms: spoken feedback through TTS following the schema order, and a text panel that presents the same content on screen.

##### 3.1.4.1 Shared system preface

All levels use the following shared preface:

System: You are a {LEVEL} *LLM-driven coaching agent* embodied as a virtual judge. Your first goal is to support public speaking practice by reducing perceived pressure while improving clarity and structure.Inputs: (a) ASR transcript; (b) eye-contact frequency time series with timestamps; (c) optional slides or outline.Constraints: Be supportive and non-humiliating; avoid medical claims; avoid diagnosis; avoid unverifiable inferences; keep feedback concise; produce valid JSON only; if evidence is insufficient, ask one brief clarification question.

##### 3.1.4.2 Output schema

All levels produce the following schema:


{ ~strengths~: [..],
~anxiety_cues_noticed~: [..],
~micro_interventions~: [..],
~content_tweak~: [..],
~next_60s_plan~: ~..~,
~one_drill~: ~..~,
~question~: ~..~ *(optional)*}


The field anxiety_cues_noticed is restricted to observable evidence, such as long pauses, filler use, or gaze distribution. It does not allow diagnostic claims about the participant's mental state. If the evidence is weak, the agent must either omit this field or phrase the observation cautiously.

##### 3.1.4.3 Level-specific constraints

Level 1 constrains output to one micro-intervention and a brief grounding cue, with a target length of 120–160 words. Level 2 allows two coaching suggestions and one guiding question, with a target length of 160–200 words. Level 3 requires one time-bounded micro-task, such as a 30-s restatement, plus one coping cue, with a target length of 120–160 words.

#### 3.1.5 Implementation and reproducibility details

The agents were implemented in Unity using Baidu's ERNIE backend for language generation. We fixed the backend model identifier, prompt templates, JSON schema, decoding parameters, and fallback logic across participants. All LLM requests and responses were logged for reproducibility and post-study auditing. To reduce nondeterminism, we used low-variance decoding and kept parameters fixed across participants, including temperature, top_p, penalty_score, and max_completion_tokens. When supported by the backend, we also set a seed for best-effort reproducibility.

The system requests structured output by enabling JSON-format responses and rejects malformed responses on the client side. For robustness, the system uses a request timeout and limited retries. If a call fails, it falls back to a short, template-based supportive message in text form and skips TTS playback. Safety constraints are enforced in the system prompt, including no diagnosis, no shaming language, no coercive content, and no claims of clinical treatment.

#### 3.1.6 Speech interaction

Speech is streamed through Unity DictationRecognizer with recognition confidence when available. The agents synthesize spoken feedback using a WaveNet-based TTS engine. In the present study, the agents did not interrupt participants during the speech. Spoken and textual feedback was delivered only in the between-round debrief segment, which reduced the risk of disrupting the speaking task or increasing pressure during performance.

#### 3.1.7 Eye-contact signal for guidance

##### 3.1.7.1 Definition and estimation

Eye-contact frequency is computed as the count of gaze hits on an avatar's face area of interest (AOI) per second, aggregated in 10-s bins:

$$\text{E}\text{C}{\text{F}}_{10s}=\frac{\#\text{on-face hits in a 10s bin}}{10}\left(\text{H}\text{z}\right)$$

We estimate the per-frame gaze target from binocular rays by using the midpoint of the shortest inter-ray segment and testing whether the point falls within a skinned-mesh face AOI. Invalid samples are discarded, and a short moving average is applied to reduce jitter. ECF is used as an auxiliary cue of attentional orientation and social engagement in VR public speaking contexts (Wechsler et al., 2021; Rubin et al., 2022). It is not treated as a direct performance score, a diagnostic anxiety measure, or a validated proxy for stable anxiety.

##### 3.1.7.2 How agents use ECF

The agents do not discretize ECF into anxiety levels and do not adapt their persona through hard thresholds. Instead, ECF is used heuristically to contextualize feedback together with the ASR transcript. For example, near-zero ECF early in the speech may prompt a gentle room-scan suggestion plus a grounding breath; spike-and-drop patterns may prompt an anchor phrase followed by a slow gaze sweep; and high-variance oscillations may prompt a three-point gaze sweep synchronized to sentence boundaries. These pattern-to-guidance mappings are transparent heuristics used for coaching, not validated inferences of anxiety. The agents are also instructed to avoid over-interpreting ECF and to phrase any gaze-related suggestion as an optional practice cue rather than as criticism.

**Figure 1:**
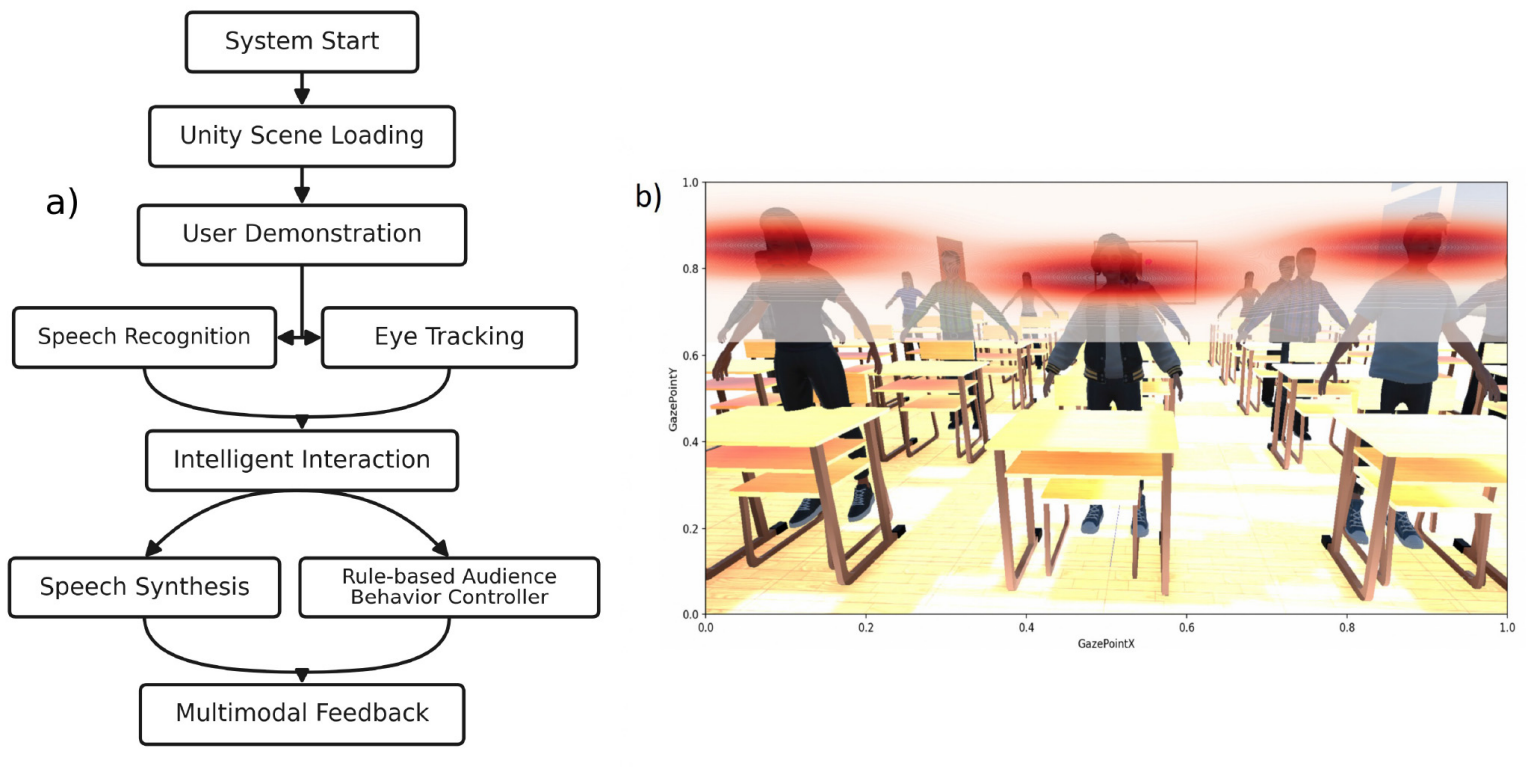
System workflow and gaze visualization. **(a)** Execution flow of the virtual agent system. Participants enter the VR classroom, deliver a speech, and generate multimodal input streams, including ASR transcript and HMD-based eye-tracking signals. These streams are passed to the reasoning layer, where the LLM-driven coaching agents generate structured feedback. Feedback is then delivered through speech synthesis, a text panel, and a rule-based audience behavior controller. **(b)** Example fixation heat map projected onto the virtual classroom view. Warmer regions indicate higher aggregated fixation density. The heat map is used to visualize gaze distribution and is not treated as a diagnostic anxiety measure.

In response to the need for stronger reporting, ECF is treated as a secondary behavioral indicator rather than as a primary outcome. Exploratory analyses relating ECF to self-reported anxiety are reported separately in the Section 5. An example visualization of the aggregated fixation heat map is shown in Figure 1b.

#### 3.1.8 Psychological safety and feedback constraints

All agents share persona definitions, a fixed output schema, conservative decoding settings, and refusal or deferral rules when evidence is insufficient. The safety constraints were designed to keep the interaction supportive and reversible.

Here, *reversible* means that each suggestion can be tried briefly and abandoned without consequence, such as taking one slow breath, adding one signpost phrase, or making one left–center–right gaze sweep. The agents do not ask participants to disclose sensitive personal experiences, do not diagnose anxiety, and do not frame nervousness as pathology. The agents also avoid coercive language, global negative judgments, and shaming statements.

Supportive nonverbal reactions, such as nods, neutral gaze, and brief applause, are generated by simple rule-based triggers. These reactions are not used to punish low ECF, increase audience hostility, or escalate social pressure after poor performance. The virtual audience never frowns, mocks the participant, or increases negative behavior in response to the participant's speech. This design is intended to preserve the social-evaluative context of public speaking while reducing the risk that feedback becomes threatening or humiliating.

#### 3.1.9 System overview and study modes

The agent pipeline integrates perception, reasoning, and feedback delivery into a closed loop, as shown in Figure 1a. During speaking, the system streams the ASR transcript and HMD-based eye-tracking signals. These inputs feed the reasoning layer, where the LLM-driven coaching agents interpret speech content together with ECF as an auxiliary cue. During the speech itself, the judges and audience maintain supportive nonverbal behavior but do not interrupt the participant. During the between-round debrief, the system delivers structured spoken and textual feedback.

The system has two delivery channels. The first is the coaching channel, which presents structured spoken and textual feedback through TTS and an on-screen text panel. The second is a rule-based audience behavior controller. This controller triggers brief nonverbal behaviors, such as nodding, gaze following, and short applause, in response to predefined session events. It does not use LLM-generated decisions and does not modify audience hostility.

In the user study, the nonverbal audience behavior controller was identical across the Experimental and Control conditions. Thus, both groups experienced the same VR classroom, the same audience layout, the same judge positions, and the same supportive nonverbal behaviors during speeches. The difference between conditions was limited to the between-round feedback segment. In the Experimental condition, feedback was generated by the LLM-driven coaching agents based on each participant's ASR transcript and delivery cues. In the Control condition, feedback followed a pre-recorded generic script with similar timing and structure but without personalization or interactive clarification. This distinction allowed the study to compare performance-contingent coaching with attention-matched generic feedback while keeping the VR exposure and nonverbal audience context stable.

### 3.2 Virtual environment design

**Figure 2:**
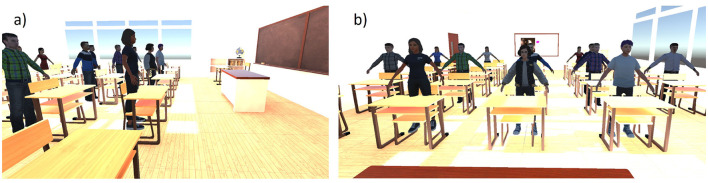
VR classroom used for the public speaking task. **(a)** The participant enters the virtual classroom from the side aisle and walks toward the podium under system guidance. This stage presents the audience before the speech begins and captures the anticipatory phase of public speaking. **(b)** The participant stands at the podium facing three virtual judges and nine audience members. The classroom layout, avatar positions, lighting, and viewpoint were kept constant across conditions and sessions.

The VR environment was developed in Unity and takes the form of a classroom-like public speaking scenario, as shown in Figure 2. Participants first enter the virtual classroom from the side aisle and approach the podium under voice guidance. After arriving at the podium, they face the virtual judges and audience before beginning the speech.

A podium is placed at the front of the room. Three virtual judges are seated in the first row, and nine audience members occupy the following rows. The three judges correspond to the three coaching roles described above: Level 1, the self-soothing coach, on the left; Level 2, the skills coach, in the center; and Level 3, the stress-inoculation coach, on the right. All judges and audience members face the podium and exhibit gaze-following behavior. The judges can additionally produce brief supportive nonverbal reactions, such as nods and applause, when triggered by predefined events.

From an interaction-design perspective, the classroom serves two purposes. First, it provides a recognizable and ecologically plausible presentation context for university students. Second, it preserves a sense of social evaluation that is central to PSA while allowing the behavior of judges and audience members to remain supportive and controllable.

When the participant enters the classroom, the system provides voice guidance from the side aisle to the podium. This stage is intended to capture the buildup before speaking, as the audience comes into view. After reaching the podium, the participant faces the audience and begins the speech. The speaking stage is designed to evoke social-evaluative pressure while keeping the task consistent across participants. To maintain experimental control, all sessions used the same classroom layout, avatar set, seating positions, viewpoints, and lighting. Only the speech topics varied across rounds and follow-up sessions, which reduced content repetition while keeping the interaction context stable.

### 3.3 Questionnaire measures

Unless otherwise stated, all questionnaire items were rated on a 5-point Likert scale, where 1 = Strongly Disagree and 5 = Strongly Agree. Internal consistency statistics for multi-item scales are reported in the Section 5.

#### 3.3.1 State public speaking anxiety

Participants rated their current level of anxiety, nervousness, or discomfort before and after each VR public speaking task using the Subjective Units of Distress Scale (SUDS), ranging from 0 = not anxious at all to 100 = extremely anxious (Wolpe and Lazarus, 1966). This measure was used to capture acute state anxiety associated with each speaking task.

#### 3.3.2 PRCA-derived public speaking anxiety appraisals

Public speaking anxiety appraisals were measured using six items adapted from the PRCA scale (McCroskey, 1978). Example items included “I feel muscle tension when speaking” and “I feel relaxed during speeches” (reverse-coded). Because these items were administered repeatedly over a short period, we interpret them as repeated self-appraisals of public speaking anxiety rather than as evidence of change in stable trait anxiety.

#### 3.3.3 Perceptions of the intelligent agent

The perceived sociability of the virtual agent was measured using 12 adjective descriptors adapted from Powers and Kiesler's social presence scale (Powers and Kiesler, 2006), such as *warm, friendly, kind*, and *trustworthy*. Interpersonal closeness was assessed using seven items that measured perceived emotional connection and distance during the interaction (Kumar and Benbasat, 2002). Sample items included “I felt close to the virtual agent” and “It felt distant during the interaction.”

Fear of being judged was measured with five items adapted from Lawlis's Fear Survey Schedule (Lawlis, 1971). Example items included “I was afraid of being criticized by the virtual agent” and “I worried about appearing foolish during the speech.”

#### 3.3.4 User experience evaluation

Participants evaluated their experience with the virtual agent across the following dimensions:

- Usefulness: four items, such as “It helped me practice speeches more effectively.”
- Ease of use: three items, such as “It was easy to operate.”
- Enjoyment: two items, such as “It was fun to use.”
- Overall satisfaction: one item.
- Willingness to continue using the system: one item.

Additionally, three open-ended questions were included:

1. In what ways was the virtual agent particularly helpful in preparing your speech?
2. What aspects did you find less useful? Why?
3. If you could redesign the agent, what features would you add or improve?

#### 3.3.5 VR presence and prior LLM experience

As a contextual measure of immersion, participants rated their sense of presence in the virtual environment on a 0–100 scale, where 0 = not realistic at all and 100 = fully immersive (Barfield and Weghorst, 1993). Participants were also asked to rate their prior experience using large language models or intelligent agents, such as ChatGPT or ERNIE Bot, over the past few months on a 7-point scale, where 1 = never used and 7 = used daily.

## 4 Experiment

To evaluate the proposed system, we conducted a two-arm randomized controlled user study with a mixed factorial design. The between-subjects factor was Group (Experimental vs. Control). Within subjects, each participant completed two speech rounds, yielding a Round (1 vs. 2) × Time (Pre vs. Post) repeated-measures structure for state anxiety. To examine whether observed benefits extended beyond the initial session, both groups also completed matched follow-up sessions at one week and one month.

**Figure 3:**
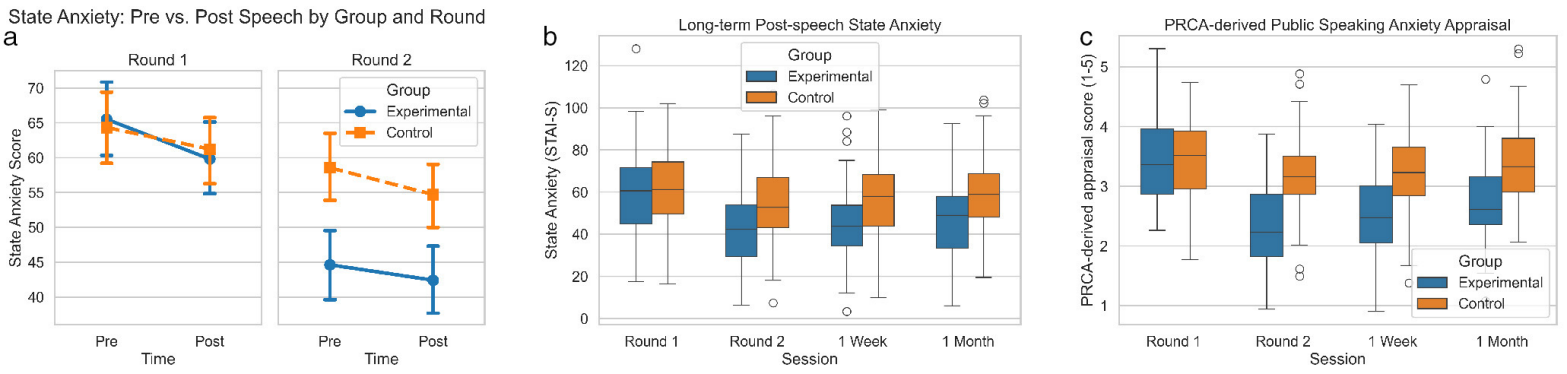
Anxiety-related outcomes by condition and session. **(a)** Pre- and post-speech state anxiety in the Experimental and Control groups for Round 1 and Round 2. Points show estimated marginal means, and error bars show 95% confidence intervals. **(b)** Post-speech state anxiety across four sessions: Round 1, Round 2, 1-week follow-up, and 1-month follow-up. **(c)** PRCA-derived public speaking anxiety appraisals across the same four sessions. In panels **(b,c)**, boxplots show the median, interquartile range, and non-outlier range.

The study was designed to evaluate not only speaking-related outcomes, but also how participants experienced the embodied *LLM-driven coaching agents* as interactive support in a socially evaluative VR setting. Table 3 summarizes the descriptive statistics for the primary anxiety-related measures, and Figure 3 visualizes the main temporal patterns across conditions and sessions.

**Table 3:** Descriptive statistics for anxiety-related measures by group (experimental vs. control).

Variable	Experimental (*n* = 60)		Control (*n* = 60)	
	Mean	SD	Mean	SD
*State anxiety*				
Pre-speech (Round 1)	65.52	21.12	64.34	20.56
Post-speech (Round 1)	59.83	20.85	61.23	20.01
Pre-speech (Round 2)	44.62	19.63	58.56	19.03
Post-speech (Round 2)	42.41	18.70	54.71	19.39
*PRCA-derived public speaking anxiety appraisals*				
After Round 1	3.48	0.74	3.43	0.71
After Round 2	2.33	0.71	3.15	0.68
*Follow-up sessions*				
Pre-speech state anxiety (1 week)	46.80	18.90	58.30	19.50
Post-speech state anxiety (1 week)	44.17	18.23	56.50	19.66
Pre-speech state anxiety (1 month)	48.25	18.20	60.10	20.10
Post-speech state anxiety (1 month)	46.05	17.20	58.20	19.97
PRCA-derived appraisal (1 week)	2.52	0.69	3.25	0.71
PRCA-derived appraisal (1 month)	2.68	0.65	3.35	0.73

### 4.1 Ethical approval

This study was approved by the Institutional Review Board of Beijing Jiaotong University. Written informed consent was obtained from all participants prior to participation. The approving committee did not issue a formal approval identifier for this study. The ethics information reported in the manuscript and in the submission metadata has been aligned to indicate that ethics approval was obtained and that written informed consent was collected.

### 4.2 Participants

Participants were recruited to match the target use case of the system, namely individuals who experience noticeable anxiety during public speaking practice. Participants were recruited through course announcements and campus mailing lists and were primarily undergraduate students at a comprehensive university. Participants came from software engineering, communication, education, management, and other undergraduate majors and received course credit plus RMB 50 equivalent for completing the laboratory session and follow-up assessments. To identify individuals with at least moderate public speaking anxiety, we administered a pre-screening item prior to participation: “I do not feel anxious when speaking in public,” rated on a 5-point Likert scale (1 = Strongly Disagree, 5 = Strongly Agree). Students scoring above 3 were excluded because such responses indicated low or minimal anxiety and thus fell outside the target population of the study.

We acknowledge that this single-item screening approach was pragmatic rather than ideal. It allowed rapid recruitment of students who reported at least some speaking-related anxiety, but it is less rigorous than screening with a validated PSA instrument. Future trials should use a validated scale, such as the PRCA or PRPSA, during eligibility screening.

The final sample consisted of 120 participants (60 female, 60 male), randomly assigned to either the Experimental group (*n* = 60) or the Control group (*n* = 60). Participants ranged in age from 18 to 30 years (*M* = 24.4, SD = 3.5). All participants reported normal or corrected-to-normal vision and no hearing impairments. All also reported prior exposure to VR. Baseline and pre-intervention equivalence checks are reported in Section 5.1.

### 4.3 Study design and conditions

The study used a randomized controlled design with one experimental condition and one attention-matched control condition. All participants completed the same number of speeches, the same assessment schedule, and the same VR classroom scenario. The key difference between conditions lay in the nature of the feedback delivered between the two speech rounds.

#### 4.3.1 Randomization and allocation

Participants were assigned to the Experimental or Control condition in a 1:1 ratio using computer-generated block randomization with a block size of 4. The sequence was generated before data collection by a researcher who was not involved in running the VR sessions. Group assignment was revealed only after eligibility screening and written informed consent had been completed. Randomization was not stratified by gender, baseline state anxiety, or prior LLM experience. Allocation was concealed using numbered sealed envelopes that were opened after consent. These details are reported to clarify the internal-validity constraints of the user study.

In the Experimental condition, participants received interactive, performance-contingent coaching generated by the embodied *LLM-driven coaching agents*. This feedback was based on the participant's own speech content and delivery cues and followed the structured coaching logic described in Section 3.1.

In the Control condition, participants received pre-recorded generic feedback of comparable duration and similar overall structure. The control feedback consisted of three judge comments matched to the Experimental feedback in delivery modality, number of segments, approximate duration, and strengths-first structure. However, this feedback was not adapted to the participant's individual speech, transcript, gaze behavior, or content, and it did not support interactive follow-up or clarification. This design allowed us to compare adaptive coaching against a control condition that matched exposure time and feedback presence, while withholding personalization and contingent interaction.

The control feedback script was:

Judge 1: You completed the first speech. It is common to feel nervous in this situation. Before your next speech, take one slow breath and focus on speaking at a steady pace.

Judge 2: Try to make your structure easy to follow. You can use a clear opening, one or two main points, and a short conclusion.

Judge 3: For the next speech, choose one key message and deliver it clearly. Remember to look around the room and pause briefly between ideas.

Feedback matching statistics are reported in Section 5.1.

### 4.4 Procedure

Each participant completed the study individually in the laboratory. At the beginning of the session, a researcher introduced the procedure and data-collection process, explaining that the participant would deliver two speeches in front of a virtual audience and evaluation system, followed by questionnaire measures.

Participants first completed the pre-speech state anxiety measure, then put on an Oculus Quest Pro headset and entered the virtual classroom. Guided by voice prompts, they walked from a side aisle to the podium and began the first speech. The first speech used Topic A: “Should university students use ChatGPT to support their learning?” Each participant was given 5 min to prepare and 2 min to speak. Immediately after the first speech, participants completed the post-speech state anxiety measure and the PRCA-derived public speaking anxiety appraisal measure.

Next, both groups remained in the same VR classroom for a feedback segment of equal duration. In the Experimental condition, the three virtual judges delivered interactive, performance-contingent coaching generated by the *LLM-driven coaching agents*. In the Control condition, the judges delivered pre-recorded generic feedback with similar timing and structure, but without personalization or interaction.

Participants then completed the second speech round. Before starting, they again completed the pre-speech state anxiety measure. The second speech used Topic B: “Should universities require a common general-education course for all undergraduates?” As in Round 1, participants were given 5 min to prepare and 2 min to speak. During the speech itself, the judges did not interrupt; instead, they maintained gaze and exhibited natural nonverbal behaviors to preserve the social-evaluative context. After the speech, participants removed the headset and completed the post-speech state anxiety measure together with the second PRCA-derived public speaking anxiety appraisal measure.

After both speech rounds, participants completed a final questionnaire assessing perceived sociability of the agents, interpersonal closeness, fear of being judged, user experience, and VR presence. An independent observer remained present throughout the session to ensure protocol compliance and minimize interference. Each laboratory session lasted approximately 35 min.

### 4.5 Follow-up sessions

To evaluate whether self-reported changes remained observable in a comparable VR practice context, both groups returned for two follow-up sessions: one at 1 week and one at 1 month after the initial laboratory session. At each follow-up, participants re-entered the same VR classroom and delivered a new speech on a non-repeated topic. The 1-week follow-up used Topic C: “Should universities require students to disclose their use of AI tools in coursework?” The 1-month follow-up used Topic D: “Should universities require a compulsory course on civic literacy and ethics?”

For each follow-up session, participants completed the pre-speech and post-speech state anxiety measures as well as the PRCA-derived public speaking anxiety appraisal measure. No additional coaching was provided to either group during the follow-ups. This design allowed us to examine whether self-reported changes observed after the initial intervention session remained observable when participants later returned to the same VR context without receiving new coaching input. Of the 120 participants randomized, all 120 completed the laboratory session, 119 completed the 1-week follow-up, and 117 completed the 1-month follow-up. At 1 week, 59 Experimental and 60 Control participants completed follow-up (two-sided Fisher's exact test, *p* = 1.000). At 1 month, 58 Experimental and 59 Control participants completed follow-up (two-sided Fisher's exact test, *p* = 1.000). Overall attrition was low: 1 participant was lost at the 1-week follow-up, corresponding to an attrition rate of 0.8% from the randomized sample, and 3 participants were lost at the 1-month follow-up, corresponding to an attrition rate of 2.5%. Group-specific attrition was also small, with 1.7% attrition in the Experimental group and 0% in the Control group at 1 week, and 3.3% in the Experimental group and 1.7% in the Control group at 1 month.

### 4.6 Measures used in the experiment

The study combined repeated anxiety-related measures with post-session assessments of agent perception and user experience. State anxiety was measured before and after each speech to capture immediate speaking-related changes. PRCA-derived public speaking anxiety appraisals were collected after each main round and at both follow-up sessions to assess short-term self-appraisals of public speaking anxiety over time. After the two main rounds, participants also evaluated the coaching agents in terms of sociability, interpersonal closeness, fear of being judged, usefulness, ease of use, enjoyment, overall satisfaction, willingness to continue using the system, and VR presence. In the present paper, these post-session measures are used to contextualize the interaction design and are complemented by open-ended responses that provide richer insight into how participants experienced the coaching process. The corresponding instruments are described in Section 3.3. Although both pre-speech and post-speech state anxiety were collected at follow-up, the longitudinal analyses reported in this paper focus on post-speech state anxiety as the primary session-level endpoint, because it most directly reflects participants' anxiety after re-entering a comparable speaking context without additional coaching. Pre-speech follow-up state anxiety is reported descriptively in Table 3 and treated as a secondary endpoint.

### 4.7 Exploratory secondary behavioral and log-based outcomes

These measures were not primary endpoints and were not blinded external performance ratings. They were included as exploratory indicators to provide preliminary converging evidence beyond self-report, and they should not be interpreted as confirmatory evidence of objective public speaking improvement.

To address the concern that self-report alone cannot establish whether the intervention affected speaking behavior, we also extracted exploratory behavioral and system-log indicators. From the HMD eye-tracking stream, we computed mean ECF, the proportion of valid gaze samples falling on judge or audience face AOIs, the proportion of near-zero ECF bins, and gaze sweep coverage across left, center, and right audience zones. From ASR transcripts, we extracted word count, estimated speech rate when timestamps were available, filler frequency, lexical diversity, and the number of explicit structure markers such as “first,” “second,” and “in conclusion.” From interaction logs, we summarized feedback duration, feedback word count, malformed JSON rejections, fallback events, TTS failures, and user clarification requests.

These measures were treated as secondary and exploratory rather than as blinded performance ratings. We used Group × Round mixed models or mixed ANOVAs for behavioral changes from Round 1 to Round 2 and examined correlations between changes in ECF and changes in self-reported state anxiety. Because the study did not include blinded external performance ratings, these analyses are interpreted as preliminary converging evidence rather than proof of objective public speaking improvement.

### 4.8 Qualitative analysis

Open-ended responses were analyzed using an inductive thematic analysis approach. Two coders independently coded all responses. The coding unit was a complete participant response to each open-ended question. The coders first reviewed 20% of responses to develop an initial codebook, then independently coded the full dataset. Inter-rater agreement was assessed using Cohen's κ and reached 0.82. Disagreements were resolved through discussion. Theme frequencies are reported by group to clarify whether comments about helpfulness, fear of judgment, realism, and speech prosody differed between the Experimental and Control conditions.

## 5 Results

Table 3 summarizes the descriptive statistics for the primary anxiety-related measures. To address our research questions, we used separate models for the complete in-laboratory repeated-measures data and the follow-up data with small amounts of attrition. For the two in-lab speech rounds, we fitted a 2 × 2 × 2 mixed ANOVA for state anxiety with *Group* (Experimental vs. Control) as a between-subjects factor and *Round* (1 vs. 2) and *Time* (Pre vs. Post) as within-subjects factors. For follow-up outcomes, we fitted linear mixed-effects models with fixed effects for *Group, Session*, and their interaction, a random intercept for participant, and all available observations. Fixed effects in these follow-up models were evaluated using Wald χ^2^ tests. Where appropriate, we performed planned simple-effects comparisons with correction for multiple testing. Statistical significance was set at *p* < 0.05.

For the in-laboratory ANOVA models, residual normality was inspected using Shapiro–Wilk tests and did not indicate substantial deviations from normality (all *p*>0.05). Given the sample size (*N* = 120), these models are also generally robust to minor departures from normality. Homogeneity of variance for the between-subjects factor (*Group*) was assessed using Levene's tests, which showed no evidence of heteroscedasticity for the primary outcomes (all *p*>0.05). For repeated-measures factors with two levels (e.g., *Time, Round*), sphericity was not an issue. The follow-up linear mixed-effects models did not require complete-case deletion or a sphericity assumption and used all available follow-up observations.

### 5.1 Preliminary reporting checks

Before testing the main research questions, we examined baseline and pre-intervention equivalence, internal consistency of multi-item scales, feedback matching, and follow-up completion. Table 4 reports group comparisons on demographic and pre-intervention variables. Baseline state anxiety was defined as Round 1 pre-speech SUDS, and pre-feedback PRCA-derived public speaking anxiety appraisal was defined as the score collected after Round 1 but before the between-round feedback segment. These checks address whether the randomized groups were comparable before adaptive coaching was delivered.

**Table 4:** Baseline and pre-intervention equivalence by group.

Variable	Experimental	Control	Test	*p*	Effect size
Age	24.5 (3.4)	24.3 (3.6)	*t*_(118)_ = 0.31	0.759	*d* = 0.06
Gender	30 female/30 male	30 female/30 male	$\chi_{\left(1\right)}^{2}=0.00$	1.000	*V* = 0.00
VR familiarity	4.12 (0.98)	4.05 (1.03)	*t*_(118)_ = 0.38	0.707	*d* = 0.07
Prior LLM experience	4.28 (1.44)	4.15 (1.38)	*t*_(118)_ = 0.50	0.616	*d* = 0.09
Screening item	2.08 (0.63)	2.13 (0.60)	*t*_(118)_ = −0.45	0.654	*d* = −0.08
Round 1 pre-speech SUDS	65.52 (21.12)	64.34 (20.56)	*t*_(118)_ = 0.31	0.757	*d* = 0.06
Pre-feedback PRCA-derived appraisal	3.48 (0.74)	3.43 (0.71)	*t*_(118)_ = 0.38	0.706	*d* = 0.07

Table 5 reports internal consistency for all multi-item measures in the present sample. Single-item measures, including overall satisfaction, willingness to continue using the system, VR presence, and prior LLM experience, were not included in reliability calculations.

**Table 5:** Internal consistency of multi-item measures.

Scale	Items	α	ω
PRCA-derived appraisal, Round 1	6	0.84	0.85
PRCA-derived appraisal, Round 2	6	0.86	0.87
PRCA-derived appraisal, 1-week follow-up	6	0.85	0.86
PRCA-derived appraisal, 1-month follow-up	6	0.87	0.88
Perceived sociability	12	0.92	0.93
Interpersonal closeness	7	0.88	0.89
Fear of being judged	5	0.83	0.84
Usefulness	4	0.86	0.87
Ease of use	3	0.79	0.80
Enjoyment	2	0.76	0.77

The feedback segment was designed to be attention-matched across conditions. As shown in Table 6, the Experimental and Control conditions are summarized on feedback duration and word count, and system logs are used to report malformed JSON rejections, fallbacks, TTS failures, and clarification requests.

**Table 6:** Feedback matching and system-log summary.

Measure	Experimental	Control
Feedback duration, seconds	96.4 (12.5)	94.8 (10.3)
Feedback word count	272.6 (31.8)	267.9 (18.4)
Number of judge segments	3	3
Malformed JSON rejections	Four total; 6.7% participants affected	Not applicable
Fallback events	Two total	Not applicable
TTS failures	One total	0
User clarification requests	11 total; *M* = 0.18 per participant	Not applicable

Follow-up completion was high. All 120 randomized participants completed the laboratory session, 119 completed the 1-week follow-up, and 117 completed the 1-month follow-up, corresponding to overall attrition rates of 0.8% and 2.5%, respectively. Attrition did not differ significantly between groups at 1 week (two-sided Fisher's exact test, *p* = 1.000) or 1 month (two-sided Fisher's exact test, *p* = 1.000), and the follow-up mixed-effects models used all available observations.

### 5.2 Effects across repeated speaking rounds

We first examined whether interactive, performance-contingent coaching was associated with different anxiety trajectories across the two in-laboratory speech rounds. As shown in Table 3 and Figure 3a, the Experimental group exhibited a markedly larger reduction from Round 1 to Round 2 than the Control group, both for pre-speech and post-speech state anxiety. Descriptively, the Experimental group decreased from *M* = 65.52 (SD = 21.12) to *M* = 44.62 (SD = 19.63) for pre-speech state anxiety, and from *M* = 59.83 (SD = 20.85) to *M* = 42.41 (SD = 18.70) for post-speech state anxiety. By contrast, the Control group showed smaller changes across rounds.

The 2 × 2 × 2 mixed ANOVA revealed a significant main effect of *Time*, *F*_(1, 118)_ = 15.28, *p* < 0.001, $\eta_{p}^{2}=0.115$, indicating an overall reduction from pre-speech to post-speech anxiety across participants. There was also a significant main effect of *Round*, *F*_(1, 118)_ = 96.79, *p* < 0.001, $\eta_{p}^{2}=0.451$, showing that anxiety was lower overall in Round 2 than in Round 1. Crucially, the *Group* × *Round* interaction was significant, *F*_(1, 118)_ = 25.58, *p* < 0.001, $\eta_{p}^{2}=0.178$, indicating that the reduction across repeated speaking rounds was larger in the Experimental group than in the Control group.

By contrast, the *Group* × *Time* interaction was not significant, *F*_(1, 118)_ = 0.06, *p* = 0.805, $\eta_{p}^{2}=0.001$, suggesting that the immediate pre-to-post shift within a given round did not differ reliably between groups. The *Round* × *Time* interaction was also not significant, *F*_(1, 118)_ = 0.75, *p* = 0.388, $\eta_{p}^{2}=0.006$, and the three-way *Group* × *Round* × *Time* interaction was not significant, *F*_(1, 118)_ = 1.78, *p* = 0.185, $\eta_{p}^{2}=0.015$. Taken together, these results suggest that the embodied *LLM-driven coaching agents* were associated primarily with greater improvement across repeated speaking practice, rather than with a differential immediate pre-to-post effect within a single speech round.

The practical size of the observed self-report change should be interpreted cautiously. The Experimental group's post-speech SUDS decreased by approximately 17 points from Round 1 to Round 2, whereas the Control group decreased by approximately 7 points. There is no universally accepted clinically meaningful threshold for SUDS change in this specific VR public speaking context, so we interpret this as a noticeable subjective reduction in speaking-related distress rather than as a clinical treatment effect. From a practical HCI perspective, this reduction suggests that adaptive coaching may help users re-enter an immediate second VR speaking attempt with noticeably lower subjective distress, which is important for iterative rehearsal systems where repeated practice is a core design goal. Planned pairwise comparisons, including mean differences, 95% confidence intervals, and pairwise effect sizes, are reported in Table 7.

**Table 7:** Planned pairwise comparisons for state-anxiety and PRCA-derived appraisal outcomes.

Contrast	Mean difference	95% CI	*p*	Effect size
Experimental Round 1 pre vs. Round 2 pre SUDS	–20.90	[–26.90, –14.90]	< 0.001	*d*_*z*_ = 0.96
Control Round 1 pre vs. Round 2 pre SUDS	–5.78	[–11.30, –0.26]	0.041	*d*_*z*_ = 0.27
Experimental Round 1 post vs. Round 2 post SUDS	–17.42	[–23.10, –11.70]	< 0.001	*d*_*z*_ = 0.82
Control Round 1 post vs. Round 2 post SUDS	–6.52	[–12.20, –0.84]	0.026	*d*_*z*_ = 0.31
Group difference Round 1 pre SUDS	1.18	[–6.40, 8.76]	0.757	*d* = 0.06
Group difference Round 2 pre SUDS	–13.94	[–20.90, –6.98]	< 0.001	*d* = −0.72
Group difference Round 1 post SUDS	–1.40	[–8.80, 6.00]	0.707	*d* = −0.07
Group difference Round 2 post SUDS	–12.30	[–19.20, –5.40]	0.001	*d* = −0.65
Group difference 1-week post SUDS	–12.33	[–19.17, –5.49]	0.001	*d* = −0.65
Group difference 1-month post SUDS	–12.15	[–18.88, –5.42]	0.001	*d* = −0.65
Group difference PRCA-derived appraisal Round 2	–0.82	[–1.07, –0.57]	< 0.001	*d* = −1.18
Group difference PRCA-derived appraisal 1 week	–0.73	[–0.98, –0.48]	< 0.001	*d* = −1.04
Group difference PRCA-derived appraisal 1 month	–0.67	[–0.92, –0.42]	< 0.001	*d* = −0.97

Negative mean differences indicate lower scores at the later time point or in the Experimental group, depending on the contrast.

### 5.3 Follow-up maintenance effects

To address RQ3, we next examined whether the differences observed during the laboratory session remained observable at follow-up in the same VR context. Both groups completed matched follow-up sessions at 1 week and 1 month. At each follow-up, participants re-entered the same VR classroom, delivered a new speech on a non-repeated topic, and completed the same anxiety-related measures. No additional coaching was provided during follow-up for either group. Figure 3b visualizes the post-speech state anxiety trajectories across Round 1, Round 2, the 1-week follow-up, and the 1-month follow-up for both groups. Figure 3c shows the corresponding pattern for PRCA-derived public speaking anxiety appraisals across the same four sessions.

#### 5.3.1 Post-speech state anxiety across sessions

For post-speech state anxiety, we fitted a linear mixed-effects model with *Group* (Experimental vs. Control), *Session* (Round 1 post, Round 2 post, 1-week post, and 1-month post), and their interaction as fixed effects and participant as a random intercept. This model used all available observations rather than deleting participants with missing follow-up data. The main effect of *Session* was significant, Wald $\chi_{\left(3\right)}^{2}=48.45$, *p* < 0.001. More importantly, the *Group* × *Session* interaction was significant, Wald $\chi_{\left(3\right)}^{2}=12.72$, *p* = 0.005, indicating different maintenance trajectories across the two groups.

Descriptively, the Experimental group showed lower post-speech state anxiety at Round 2 (*M* = 42.41, SD = 18.70) than at Round 1 (*M* = 59.83, SD = 20.85), and this lower range remained observable at both follow-ups (1 week: *M* = 44.17, SD = 18.23; 1 month: *M* = 46.05, SD = 17.20). In contrast, the Control group remained closer to its Round 1 post-speech level, with follow-up values of *M* = 56.50 (SD = 19.66) at 1 week and *M* = 58.20 (SD = 19.97) at 1 month. Corrected *post hoc* comparisons indicated that, in the Experimental group, Round 2 and both follow-up sessions were significantly lower than Round 1, whereas the Control group did not show reliable follow-up reductions relative to Round 1. Pre-speech follow-up state anxiety is reported as a secondary endpoint using the values added to Table 3.

#### 5.3.2 PRCA-derived public speaking anxiety appraisals across sessions

We then fitted the same *Group* × *Session* linear mixed-effects model for PRCA-derived public speaking anxiety appraisals across Round 1, Round 2, 1 week, and 1 month. The main effect of *Session* was significant, Wald $\chi_{\left(3\right)}^{2}=123.75$, *p* < 0.001. The *Group* × *Session* interaction was also significant, Wald $\chi_{\left(3\right)}^{2}=51.90$, *p* < 0.001.

Descriptively, the Experimental group showed a lower score at Round 2 (*M* = 2.33, SD = 0.71) than at Round 1 (*M* = 3.48, SD = 0.74), with follow-up values of *M* = 2.52 (SD = 0.69) at 1 week and *M* = 2.68 (SD = 0.65) at 1 month. The Control group showed higher follow-up values (*M* = 3.25, SD = 0.71 at 1 week; *M* = 3.35, SD = 0.73 at 1 month). Overall, lower anxiety-related self-appraisals remained observable over both follow-up intervals in the Experimental group, whereas the Control group remained at a comparatively higher level. Because these scores were collected repeatedly over a short period, we interpret them as PRCA-derived self-appraisals rather than as evidence of change in stable trait anxiety.

### 5.4 Exploratory secondary behavioral and log-based analyses

We next examined gaze, transcript-derived, and log-based indicators as exploratory secondary outcomes. These analyses were intended to provide preliminary converging evidence beyond self-report, but the resulting tests should be interpreted descriptively rather than as confirmatory evidence of objective performance improvement. The results are summarized in Table 8.

**Table 8:** Exploratory secondary behavioral and log-based outcomes.

Outcome	Experimental change	Control change	Group × round/test	Interpretation
Mean ECF	+0.16 Hz	+0.05 Hz	*F*_(1, 118)_ = 9.84, *p* = 0.002, $\eta_{p}^{2}=0.077$	Larger gaze-to-face increase in Experimental group
Judge/audience face AOI proportion	+14 percentage points	+5 percentage points	*F*_(1, 118)_ = 8.92, *p* = 0.003	More face-AOI gaze
Near-zero ECF bins	–13 percentage points	–5 percentage points	*F*_(1, 118)_ = 6.78, *p* = 0.010	Fewer near-zero gaze-to-face bins
Left-center-right gaze sweep coverage	+22 percentage points	+8 percentage points	*F*_(1, 118)_ = 10.41, *p* = 0.002	More distributed gaze
Transcript word count	+36 words	+13 words	*F*_(1, 118)_ = 5.92, *p* = 0.016	Longer second speech
Estimated speech rate	+18 wpm	+6 wpm	*F*_(1, 118)_ = 4.87, *p* = 0.029	Higher speech-rate proxy
Filler frequency	–3.7 fillers/min	–1.4 fillers/min	*F*_(1, 118)_ = 5.35, *p* = 0.022	Fewer disfluencies
Structure markers	+3.5 markers	+1.0 marker	*F*_(1, 118)_ = 12.64, *p* < 0.001	More transcript-detected structure markers

To examine whether the gaze cue used by the agents was meaningfully related to anxiety in this sample, we correlated ECF with self-reported SUDS at corresponding speech rounds and correlated change in ECF with change in SUDS from Round 1 to Round 2. ECF was negatively correlated with concurrent SUDS [*r* = −0.31, 95% CI (−0.45, −0.14), *p* = 0.001], and increases in ECF from Round 1 to Round 2 were associated with larger SUDS reductions [*r* = −0.36, 95% CI (−0.50, −0.19), *p* < 0.001]. Change in left-center-right gaze sweep coverage also correlated with SUDS reduction [*r* = −0.28, 95% CI (−0.43, −0.10), *p* = 0.003]. ECF is therefore interpreted as an auxiliary behavioral indicator of attentional orientation, not as a validated diagnostic anxiety measure.

### 5.5 Agent perception and user experience

Because RQ2 concerns users' experience of the embodied agents, we compared groups on perceived sociability, interpersonal closeness, fear of being judged, usefulness, ease of use, enjoyment, overall satisfaction, willingness to continue, and VR presence. Table 9 reports descriptive statistics, corrected group comparisons, and effect sizes. Particular attention was given to fear of being judged and perceived sociability because these measures directly address whether personalized coaching changed the way participants experienced the judge-like agents.

**Table 9:** Agent perception and user-experience comparisons by group.

Measure	Experimental	Control	Test/95% CI	*p*	*d*
Perceived sociability	4.18 (0.55)	3.74 (0.62)	*t* = 4.11; CI [0.23, 0.65]	< 0.001	0.75
Interpersonal closeness	3.82 (0.61)	3.39 (0.68)	*t* = 3.64; CI [0.20, 0.66]	< 0.001	0.66
Fear of being judged	2.10 (0.72)	2.63 (0.79)	*t* = −3.84; CI [–0.80, –0.26]	< 0.001	–0.70
Usefulness	4.25 (0.50)	3.68 (0.62)	*t* = 5.55; CI [0.37, 0.77]	< 0.001	1.01
Ease of use	4.31 (0.48)	4.20 (0.51)	*t* = 1.22; CI [–0.07, 0.29]	0.226	0.22
Enjoyment	4.07 (0.60)	3.82 (0.66)	*t* = 2.17; CI [0.02, 0.48]	0.033	0.40
Overall satisfaction	4.18 (0.63)	3.75 (0.70)	*t* = 3.54; CI [0.19, 0.67]	< 0.001	0.64
Willingness to continue	4.05 (0.71)	3.60 (0.78)	*t* = 3.30; CI [0.18, 0.72]	0.001	0.60
VR presence	78.6 (12.4)	77.2 (13.1)	*t* = 0.60; CI [–3.2, 6.0]	0.550	0.11

### 5.6 Qualitative feedback from open-ended responses

To better understand participants' experience with the *LLM-driven coaching agents*, we conducted a thematic analysis of responses to three open-ended questions on helpful aspects, limitations, and desired improvements, following the coding procedure described in Section 4.8. Table 10 summarizes the main themes, group frequencies, and representative quotations.

**Table 10:** Qualitative themes by group.

Theme	Experimental *n*/%	Control *n*/%	Example quotation	Interpretation
Reduced pressure	36/60 (60%)	25/60 (42%)	“It was enthusiastic and made me feel less pressured than a real person.”	Supportive judge framing reduced threat.
Actionable feedback	41/60 (68%)	19/60 (32%)	“The suggestions gave me something concrete to try next.”	Personalized feedback was perceived as more actionable.
Lower fear of judgment	29/60 (48%)	16/60 (27%)	“I felt evaluated, but not attacked or embarrassed.”	Evaluation felt less punitive.
Limited realism	22/60 (37%)	25/60 (42%)	“The appearance is not realistic enough.”	Avatar realism limited social believability.
Stiff speech prosody	18/60 (30%)	23/60 (38%)	“The voice sounds stiff.”	TTS prosody needs improvement.
Richer gestures and interaction	24/60 (40%)	21/60 (35%)	“Include multiple types of interaction.”	Users wanted richer embodied behavior.

#### 5.6.1 Perceived effectiveness of the LLM-driven coaching agents

In the Experimental group, 36 of 60 participants (60%) described the agents as helpful because they reduced stress while still offering useful feedback. In the Control group, 25 of 60 participants (42%) made similar comments. Several participants noted that being evaluated by the agents felt less pressuring than being judged by real people. As one participant stated, “It was enthusiastic and made me feel less pressured than a real person.”

Others valued the chance to practice in a low-pressure setting while still receiving guidance. For example, one participant wrote, “It lets me practice my speech,” and another commented, “The feedback was fair.” These responses suggest that the agents supported both emotional comfort and speech practice. Actionable-feedback comments were more frequent in the Experimental condition (41/60, 68%) than in the Control condition (19/60, 32%), consistent with the personalized and performance-contingent nature of the coaching feedback.

#### 5.6.2 Perceived limitations

The most common limitation concerned realism. Participants noted that the agents' appearance and voice did not feel natural enough, which reduced the sense of speaking to a real audience or panel. One participant remarked, “The appearance is not realistic enough,” and another added, “The voice sounds stiff.” Comments about stiff prosody were mentioned by 18 of 60 Experimental participants (30%) and 23 of 60 Control participants (38%). This group-level reporting is important because both groups heard judge feedback, but only the Experimental group received personalized LLM-generated coaching. These comments suggest that limited technical realism may weaken the perceived authenticity of the interaction regardless of feedback type.

#### 5.6.3 Suggestions for future improvement

Participants mainly asked for more natural and varied interaction. Suggestions included richer gestures, more interaction types, more realistic visual design, and more natural speech prosody. As one participant put it, “Add more gestures,” while another wrote, “Include multiple types of interaction.” Another simply said, “Make it more realistic.”

Overall, participants found the *LLM-driven coaching agents* helpful and supportive, but also wanted greater realism and human-likeness. Improving interactivity and realism may strengthen both engagement and perceived training value.

## 6 Discussion

### 6.1 Effectiveness of agent feedback (RQ1)

The Experimental group showed a larger decline in self-reported anxiety from Round 1 to Round 2 than the Control group. In the 2 × 2 × 2 mixed repeated-measures ANOVA, the significant *Group* × *Round* interaction indicates that feedback from the *LLM-driven coaching agents* was associated with greater improvement across rounds than attention-matched, pre-recorded generic feedback. By contrast, the *Group* × *Time, Round* × *Time*, and three-way interactions were not significant. This suggests that the main between-group difference lay in change across repeated speaking rounds rather than in a stronger pre-to-post reduction within a single round.

The lack of a stronger *Group* × *Time* effect is informative. Post-speech SUDS may still capture residual arousal from the just-completed performance, whereas the Round 2 pre-speech assessment may better reflect anticipatory reappraisal after participants had received feedback and had time to prepare using the suggested strategies. Thus, the pattern is more consistent with feedback supporting the next speaking attempt than with an immediate condition-specific reduction during the same speech. This interpretation is consistent with prior work showing that supportive agent-based interventions can benefit student users (Rasouli et al., 2024). However, the present data do not directly test whether reduced anxiety was caused by supportive language, personalization, perceived sociability, gaze-based feedback, or novelty. The most conservative interpretation is that the combined adaptive coaching package was associated with larger self-reported anxiety reductions than generic feedback delivered for a comparable duration in the same VR setting.

### 6.2 User experience and perceived sociability (RQ2)

Participants' written comments and post-session ratings suggest that the agents were experienced as supportive rather than punitive. This is notable given that the agents were visually framed as judges within an evaluative VR setting. The Experimental group reported higher perceived sociability and lower fear of being judged than the Control group, suggesting that personalized coaching may have softened the threat of evaluation while preserving social-evaluative realism. At the same time, VR presence did not differ significantly between groups, which is consistent with the fact that both groups experienced the same embodied judges and supportive nonverbal behavior.

The findings also connect to broader theories of social presence and self-efficacy. Embodied agents may be useful in anxiety-aware practice when they are perceived as socially present, warm, and non-punitive. Strengths-first feedback and short rehearsable drills may support self-efficacy by giving speakers a specific next action rather than a global evaluation of competence. At the same time, participants did not regard the experience as fully realistic. The most common concerns involved avatar appearance and speech prosody, both of which affected how natural the interaction felt. Future versions should improve visual fidelity, speech prosody, and interaction variety so that the agents feel more credible as social partners while preserving the supportive character of the system. The practical implication is therefore not that the system produces a clinical treatment effect, but that strengths-first, performance-contingent feedback may make repeated VR public speaking practice feel more manageable and actionable for users who experience speaking-related anxiety.

### 6.3 Sustainability of intervention effects (RQ3)

The significant *Group* × *Session* interactions for post-speech state anxiety and PRCA-derived public speaking anxiety appraisals show that the two groups followed different self-report patterns over time. Lower self-reported anxiety remained observable in the Experimental group at one week and one month. These follow-up findings should not be interpreted as evidence of real-world transfer. We did not test whether participants experienced reduced anxiety in real classrooms, interviews, examinations, workplace presentations, or live-audience speaking situations. Therefore, the present results support only maintenance of self-reported improvement within the same VR practice context. Participants repeatedly entered the same VR classroom, used the same equipment, and completed comparable speaking tasks. Repeated exposure, habituation to the VR environment, and familiarity with the procedure may therefore have contributed to the observed follow-up pattern.

The PRCA-derived appraisal findings should also be interpreted cautiously. Because the adapted items were administered repeatedly over a short time window, they are better understood as short-term self-appraisals of public speaking anxiety or confidence than as measures of stable trait anxiety. Future work should test longer intervention schedules, include validated screening instruments, and examine whether gains persist in new VR contexts and real-world speaking settings.

### 6.4 Alternative explanations and mechanisms

Alternative explanations should be considered. First, repeated exposure to the same VR classroom may have produced habituation. Second, participants may have become more familiar with the headset, podium, and procedure over time. Third, the adaptive LLM interaction may have produced novelty or demand effects, especially because participants knew that they were receiving a new AI coaching system. Fourth, personalization, embodiment, supportive language, and gaze-related suggestions were bundled together in the Experimental condition, so the present two-group design cannot isolate their separate effects.

Future studies should include additional comparison groups, such as non-LLM personalized feedback, LLM feedback without embodiment, embodied generic feedback, and VR exposure without feedback. Such designs would help determine whether the observed self-report differences are driven mainly by personalization, LLM novelty, embodied social presence, repeated exposure, or specific coaching strategies.

## 7 Limitations

Several limitations should be noted. First, the sample consisted primarily of university students, limiting generalizability beyond student populations and classroom-like speaking tasks. Second, the primary outcomes relied on self-report. Although we added exploratory gaze, transcript-derived, and log-based analyses, the study did not include physiological measures such as heart rate, electrodermal activity, or cortisol, and did not include blinded external ratings of public speaking performance. Third, the single-item screening procedure was less rigorous than using a validated PSA screening instrument. Fourth, although allocation was concealed using numbered sealed envelopes, participants and experimenters were not blinded to condition after assignment because the feedback formats differed. Fifth, repeated exposure to the same VR classroom may have contributed to lower anxiety at follow-up, and the study did not test transfer to real classrooms, interviews, examinations, workplace presentations, or live-audience public speaking. Accordingly, no claim is made that the observed self-reported reductions generalize to real-world public speaking performance or anxiety outside the VR practice setting. Sixth, novelty and demand characteristics associated with adaptive LLM feedback may have influenced participants' responses. Finally, the present study did not test whether self-reported anxiety reductions translate into better real-world public speaking performance, improved vocal prosody, greater persuasiveness, or stronger audience-rated communication quality.

## 8 Conclusion

This paper presented the design and evaluation of embodied *LLM-driven coaching agents* for VR public speaking practice. The system combines immersive classroom-based speaking scenarios with supportive, structured, and performance-contingent feedback designed to balance social-evaluative realism with psychological safety. Compared with an attention-matched Control condition receiving pre-recorded generic feedback, the Experimental condition was associated with larger reductions in self-reported state anxiety across repeated speaking rounds, and these benefits were largely maintained at 1-week and 1-month follow-up.

Beyond speaking-related outcomes, the study contributes an HCI perspective on how embodied AI agents can function as coaching interfaces in stressful interactive settings. The findings suggest that adaptive feedback may be most useful when it keeps evaluative realism while offering supportive framing, concise interventions, and immediate rehearsable actions. At the same time, participants' qualitative feedback highlights an important next step for future work: increasing realism, prosody, and behavioral richness so that embodied AI coaching feels not only helpful, but also more natural and socially believable.

Overall, the findings suggest that VR combined with *LLM-driven coaching agents* is a promising direction for scalable, human-centered public speaking support. The system should not be viewed as a replacement for clinical intervention, but it may serve as a scalable, low-pressure, repeatable practice aid for users who experience speaking-related anxiety. We did not test transfer to real classrooms, interviews, examinations, or live-audience speaking situations; therefore, no real-world transfer claim is made. Future work should examine more diverse participant populations, longer intervention schedules, richer multimodal sensing, and more expressive embodied behaviors. It should also test whether reductions in self-reported anxiety are accompanied by externally rated improvements in public speaking performance, including clarity, persuasiveness, charisma, fluency, and vocal prosody. Such work would clarify whether anxiety reduction functions as a pathway toward better real-world communication rather than an endpoint in itself.

## Data Availability

The raw data supporting the conclusions of this article will be made available by the authors, without undue reservation.
